# Modulation of the immune microenvironment of high-risk ductal carcinoma in situ by intralesional pembrolizumab injection

**DOI:** 10.1038/s41523-021-00267-z

**Published:** 2021-05-25

**Authors:** Alexa C. Glencer, Jasmine M. Wong, Nola M. Hylton, Gregor Krings, Emma McCune, Harriet T. Rothschild, Tristan A. Loveday, Michael D. Alvarado, Laura J. Esserman, Michael J. Campbell

**Affiliations:** 1 Department of Surgery, University of California San Francisco, San Francisco, CA USA; 2 Department of Pathology, University of California San Francisco, San Francisco, CA USA; 3 University of California San Francisco School of Medicine, San Francisco, CA USA

**Keywords:** Tumour immunology, Breast cancer, Phase I trials

## Abstract

Ductal carcinoma in situ (DCIS) is a risk factor for the subsequent development of invasive breast cancer. High-risk features include age <45 years, size >5 cm, high-grade, palpable mass, hormone receptor negativity, and HER2 positivity. We have previously shown that immune infiltrates are positively associated with these high-risk features, suggesting that manipulating the immune microenvironment in high-risk DCIS could potentially alter disease progression. Patients with high-risk DCIS were enrolled in this 3 × 3 phase 1 dose-escalation pilot study of 2, 4, and 8 mg intralesional injections of the PD-1 immune checkpoint inhibitor, pembrolizumab. Study participants received two intralesional injections, three weeks apart, prior to surgery. Tissue from pre-treatment biopsies and post-treatment surgical resections was analyzed using multiplex immunofluorescence (mIF) staining for various immune cell populations. The intralesional injections were easily administered and well-tolerated. mIF analyses demonstrated significant increases in total T cell and CD8^+^ T cell percentages in most patients after receiving pembrolizumab, even at the 2 mg dose. T cell expansion was confined primarily to the stroma rather than within DCIS-containing ducts. Neither cleaved caspase 3 (CC3) staining, a marker for apoptosis, nor DCIS volume (as measured by MRI) changed significantly following treatment. Intralesional injection of pembrolizumab is safe and feasible in patients with DCIS. Nearly all patients experienced robust total and CD8+ T cell responses. However, we did not observe evidence of cell death or tumor volume decrease by MRI, suggesting that additional strategies may be needed to elicit stronger anti-tumor immunity.

## Introduction

DCIS is a non-obligate precursor of invasive breast cancer characterized by confinement of proliferating ductal cells within the basement membrane of mammary ducts. The rate and latency of progression from DCIS to invasive breast cancer in the absence of treatment are unknown. DCIS itself is not a lethal condition, but women with DCIS are at higher risk of developing subsequent local or metastatic invasive breast cancer.

DCIS is not one condition but, rather, a spectrum of disease, ranging from indolent lesions more akin to markers of increased risk for hormone-receptor-positive invasive breast cancer with minimal associated mortality to true precursors of triple-negative or HER2 positive invasive cancers that are likely to arise in a one-year to a two-year timeframe with significant associated metastatic potential. A recent evaluation of 100,000 cases of DCIS revealed that there is a small group of women who develop the metastatic disease without first developing an invasive cancer recurrence^1^. Certain biological factors, including tumor grade, comedonecrosis, tumor size, hormone receptor-negative status, and HER2 positive status, are known to predict a greater risk of disease recurrence or progression but do not accurately identify the group of patients at risk of death from their DCIS and do not predict the therapy that could prevent that event ^2^.

The importance of intact immune surveillance in controlling neoplastic transformation has been known for decades. The tumor immune microenvironment, consisting of multiple cellular and molecular factors, has an essential role in the biological behavior of cancer^3^. Accumulating evidence shows a correlation between tumor-infiltrating lymphocytes (TILs) in cancer tissue and favorable prognosis in various malignancies. In particular, the presence of CD8^+^ T cells and the ratio of CD8^+^ effector T cells/Foxp3^+^ regulatory T cells seems to correlate with improved prognosis and long-term survival in many solid tumors. Conversely, regulatory T cells (Tregs) and myeloid-derived suppressor cells (MDSCs) have been found to aggregate in the tumor immune microenvironment, suppressing immune responses and inducing angiogenesis, thus facilitating cancer growth and inhibiting the effectiveness of immunotherapy ^4,5^.

Little is known about the behavior of these immune cell populations in premalignant lesions. Studies have shown that high-grade, hormone receptor (HR)-negative invasive breast cancers have a greater inflammatory component (significant macrophage and T cell infiltration) than low-grade, HR-positive tumors^6–10^. Importantly, tumor-infiltrating macrophages are associated with early recurrence in HR-negative invasive breast cancer. High-grade DCIS is also characterized by an increased population of macrophages detected as CD68 positive cells using immunohistochemistry (IHC) ^11–13^.

A recent study conducted by our group sought to characterize the immune microenvironment of DCIS^2^. In a comparison of 53 cases of high-grade DCIS, enriched for large lesions, to 65 cases of low-grade DCIS, immune infiltrates were correlated with high-risk DCIS features (high Van Nuys Prognostic Index, palpability, high-grade, comedonecrosis, high Ki67, HR-negative, and HER2-positive). No single immune cell population was associated with prognostic value. However, combinations of immune cell populations, specifically those that included low numbers of activated CD8^+^ T cells and high numbers of CD115^+^ macrophages, were associated with a high risk for recurrence. These results suggest that activating CD8^+^ T cells within DCIS lesions could potentially alter disease progression.

PD-1 (programmed death receptor-1) is an Ig superfamily member related to CD28 and CTLA-4 that has been shown to negatively regulate antigen receptor signaling upon engagement of its ligands (PD-L1 and/or PD-L2)^14,15^. PD-1 is expressed on activated lymphocytes including CD4^+^ and CD8^+^ T cells, B cells, Tregs, and natural killer cells. PD-L1 is expressed on a variety of hematopoietic cells (macrophages, dendritic cells, T cells, B cells, NK cells), as well as various non-hematopoietic cells such as epithelial and endothelial cells. A variety of cancers have been found to express abundant levels of PD-L1, and evidence suggests that the PD-1/PD-L1 pathway plays a critical role in tumor immune evasion. This has led to the development of a number of therapeutic agents targeting the PD-1/PD-L1 interaction.

Pembrolizumab is a humanized anti-PD-1 monoclonal antibody (mAb) designed to block the interaction between PD-1 and its ligands. Pembrolizumab has been approved in the United States to treat patients with a variety of cancers including melanoma, non-small cell lung cancer, head and neck carcinoma, gastric cancer, esophageal cancer, cervical cancer, and cancers with high microsatellite instability. Among patients with high-risk breast cancer, pembrolizumab in combination with standard chemotherapy has been shown to nearly triple the chance of complete pathologic response in the neoadjuvant setting relative to standard chemotherapy alone ^16,17^.

In this pilot study, patients with high-risk DCIS were treated with local (intralesional) administration of pembrolizumab. The primary objective of this trial was to establish the safety and feasibility of intralesional immunotherapy in patients with DCIS. A secondary objective was to evaluate changes in the tumor immune microenvironment pre-therapy versus post-therapy. Exploratory objectives included an evaluation of tumor volume measured using MRI pre-pembrolizumab versus post-pembrolizumab therapy, as well as an evaluation of markers of apoptosis (cleaved caspase 3) and proliferation (Ki67), pre-therapy versus post-therapy.

## Results

### Patient demographics

Nine patients were enrolled in this dose-escalation phase I pilot study of intralesional pembrolizumab. The average age of these patients was 49 years (range 31–68) with an average BMI of 23 (range 19.0–30.0). All nine patients included in the study were Caucasian. Four patients were premenopausal and five patients were postmenopausal at the time of pembrolizumab treatment (Table 1).

**Table 1:** Clinical characteristics of the patient cohort.

Patient	Age (years)	Body mass index (BMI)	Menopausal status	DCIS size pre-therapy (MRI)	DCIS size post-therapy (surgical path)	Microinvasive disease at resection	Hormone receptor status	HER2 status
1	45	22.9	Pre	9.8 cm	8 cm	No	Positive	Unknown
2	61	30.0	Post	No abnormality	1.8 cm	No	Negative	Negative
3	34	19.0	Pre	3.3 cm	5.1 cm	Yes	Positive	Positive
4	42	25.7	Pre	1.1 cm	0.9 cm	No	Positive	Unknown
5	54	21.0	Post	7.8 cm	4.5 cm	Yes	Negative	Positive
6	54	20.7	Post	6.7 cm	4.5 cm	Yes	Negative	Negative
7	31	22.0	Pre	4.1 cm	3.0 cm	Yes	Positive	Negative
8	68	19.6	Post	3.7 cm	3.6 cm	No	Positive	Positive
9	50	25.3	Post	4.5 cm	4.4 cm	Yes	Positive	Positive

Hormone receptor-positive = either estrogen receptor or progesterone receptor-positive.

Hormone receptor-negative = neither estrogen receptor nor progesterone receptor-positive.

### DCIS specimen characterization

All patients had high-grade DCIS determined at the time of core biopsy (vacuum-assisted core biopsies permitted), as specified by the eligibility criteria. No patients had microinvasive disease on core biopsy, but 5 of 9 patients were subsequently found to have microinvasive disease in the setting of DCIS within their surgical resection specimens. Excluding the one patient who did not have radiographic evidence of DCIS, the average radiographic size of DCIS on pre-therapy MRI was 5.1 cm as defined by the longest dimension of non-mass enhancement (NME). The average pathologic size at the time of surgical resection, defined by span of contiguous DCIS, was 4.03 cm. 67% (6/9) patients had hormone receptor-positive (HR+) disease based upon their pre-therapy core biopsies. 44% (4/9) patients had HER2+ disease based upon analysis of their surgical specimens; core biopsy results were not used to assess HER2 status, as routine HER2 testing was not performed on these specimens (Table 1). Further pathologic characteristics pertaining to the core biopsy specimens are included in Table 2.

**Table 2:** Pathologic characteristics of core biopsy specimens.

Patient	Vacuum-assisted?	# of cores analyzed	Cores fragmented?	Lymphocytic infiltrate?
1	Yes	2	Yes	No
2	Yes	1	Yes	No
3	No	2	Yes	Yes
4	No	1	No	No
5	Yes	2	No	Yes
6	Yes	2	Yes	No
7	No	4	Yes	No
8	No	3	No	No
9	Yes	2	Yes	No

### Intralesional anti-PD-1 increased infiltration of total T cells and cytotoxic CD8^+^ T cells

**Fig. 1:**
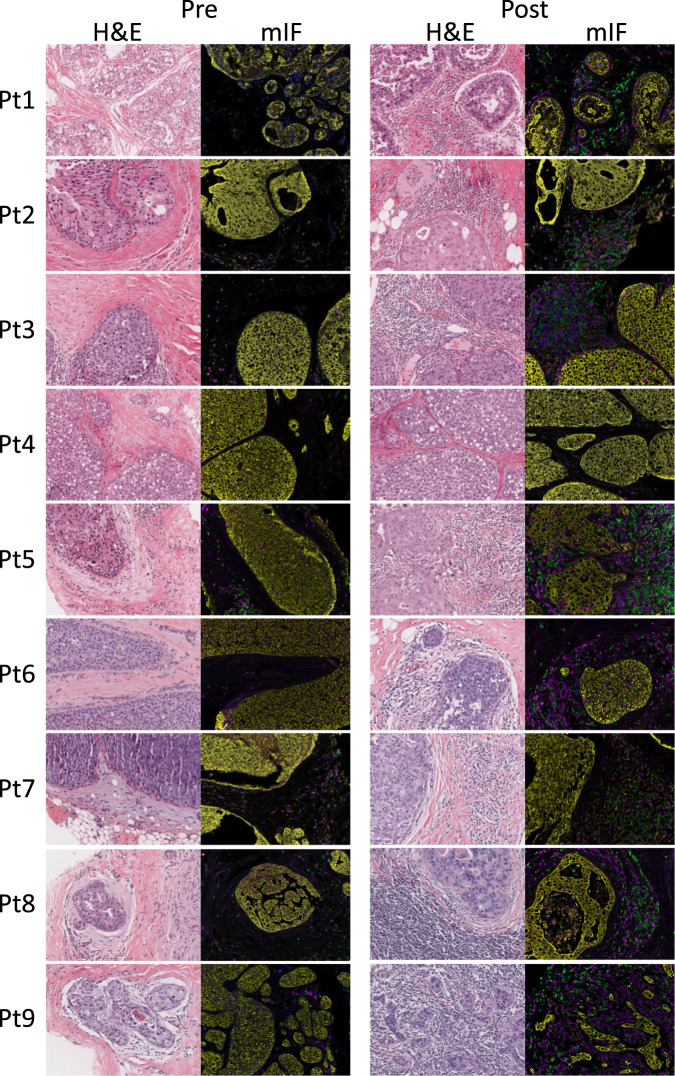
T cell infiltrates in DCIS pre-treatment and post-treatment with intralesional anti-PD-1. Representative hematoxylin and eosin (H&E) and multiplex immunofluorescence (mIF) images from pre-therapy biopsies and post-therapy surgical specimens are shown for each patient. For the mIF staining, CD3-positive cells are pseudocolored green, CD8-positive cells are magenta, and cytokeratin-positive cells are yellow. Original magnification: ×20.

**Fig. 2:**
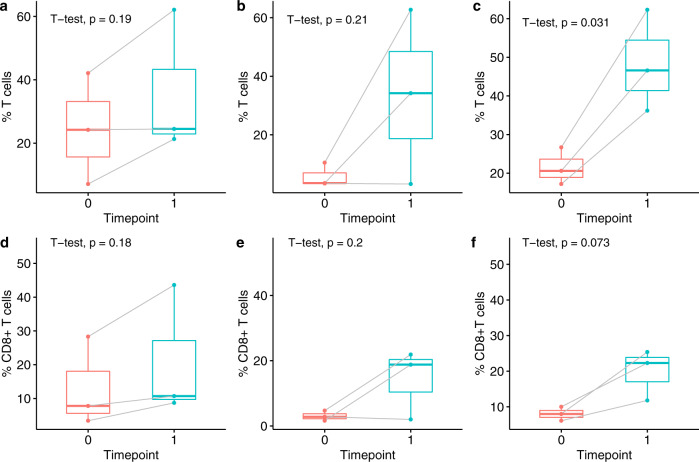
T cell infiltrates increased in 7 of 9 cases across all three dose cohorts. Total T cell and CD8^+^ T cell density in response to 2 mg pembrolizumab (**a**, **d**), 4 mg pembrolizumab (**b**, **e**), and 8 mg pembrolizumab (**c**, **f**). Timepoint 0 (pre-therapy) and timepoint 1 (post-therapy) are shown. The bounds of each box represent the interquartile range (IQR). The horizontal line within a box is the median. Whiskers extend to the largest and smallest values no further than 1.5*IQR. Outlying points beyond the ends of the whiskers are plotted individually. Each pair of connected points represents one patient’s data; *p*-values are derived from paired *t*-tests.

We evaluated changes in the DCIS immune microenvironment in response to intralesional anti-PD-1 immunotherapy using multiplex immunofluorescence staining and image analysis. Figure 1 shows representative hematoxylin and eosin (H&E) and multiplex immunofluorescence (mIF) images from pre-therapy biopsies and post-therapy surgical specimens for each patient in the study (larger images are shown in Supplementary Figs. 1–9). As can be seen, there were therapy-associated increases in T cells for most patients. Fig. 2 shows the change in T cells for each case in each dose cohort. Seven of the 9 cases demonstrated an increase in T cells post-therapy. No change in T cell percentages was seen in patient 2 in the low dose cohort (24% pre and 24% post) and patient 4 in the intermediate dose cohort (3% pre and 3% post).

**Fig. 3:**
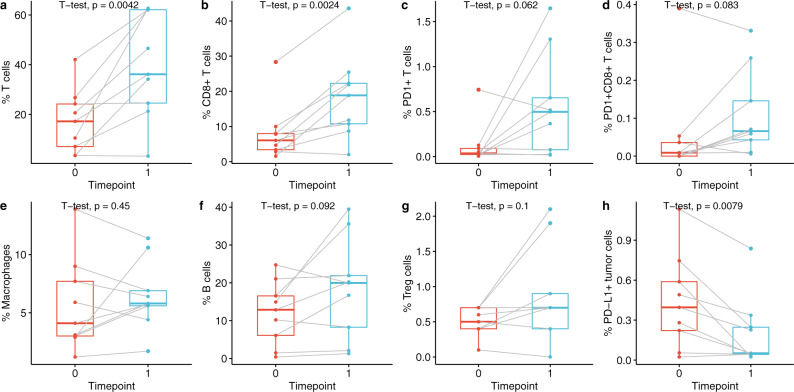
Changes in immune cell populations in response to intralesional anti-PD-1 therapy. Cell densities pre-therapy (timepoint 0) and post-therapy (timepoint 1) are shown for (**a**) T cells, (**b**) cytotoxic T cells, (**c**) PD-1^+^ T cells, (**d**) PD-1^+^ cytotoxic T cells, (**e**) macrophages, (**f**) B cells, (**g**) regulatory T cells, and (**h**) PD-L1^+^ tumor cells. The bounds of each box represent the interquartile range (IQR). The horizontal line within a box is the median. Whiskers extend to the largest and smallest values no further than 1.5*IQR. Outlying points beyond the ends of the whiskers are plotted individually. Each pair of connected points represents one patient’s data; *p*-values derived from paired *t*-tests.

Across the cohort of nine patients, the density of CD3^+^ T cells was significantly higher in post-therapy tissue [median percent of positive cells (range): 17.2 (3.4–42.1) versus 36.2 (3.2–62.7), *p* = 0.0042] (Fig. 3a). The density of CD8^+^ T cells also significantly increased post-therapy [median percent of positive cells (range): 6.1 (1.6–28.3) versus 18.8 (2.0–43.6), *p* = 0.0024] (Fig. 3b). In contrast, the density of B cells (Fig. 3f), macrophages (Fig. 3e), and Foxp3^+^ regulatory T cells (Treg)(Fig. 3g) did not significantly change in response to anti-PD-1 therapy. There was an increase in PD-1^+^ T cells and PD-1^+^ CD8^+^ T cells in 5 of the 9 cases post-therapy, but this was not significant across the whole cohort (Fig. 3c, d). Finally, the density of PD-L1^+^ tumor cells significantly decreased post-therapy [median percent of positive cells (range): 0.4 (0.02–1.13) versus 0.05 (0.02–0.84), *p* = 0.0079] (Fig. 3h), whereas the density of PD-L1^+^ immune cells was not significantly altered (data not shown).

To assess T cell activation, we examined the intracellular expression of Ki-67, a cell-cycle marker expressed by cycling or recently divided cells. The density of Ki67^+^ T cells did not significantly change pre-therapy to post-therapy [median percent of positive cells (range): 0.86 (0.02–2.2) versus 0.84 (0.05–3.68), *p* = 0.26] (data not shown).

**Fig. 4:**
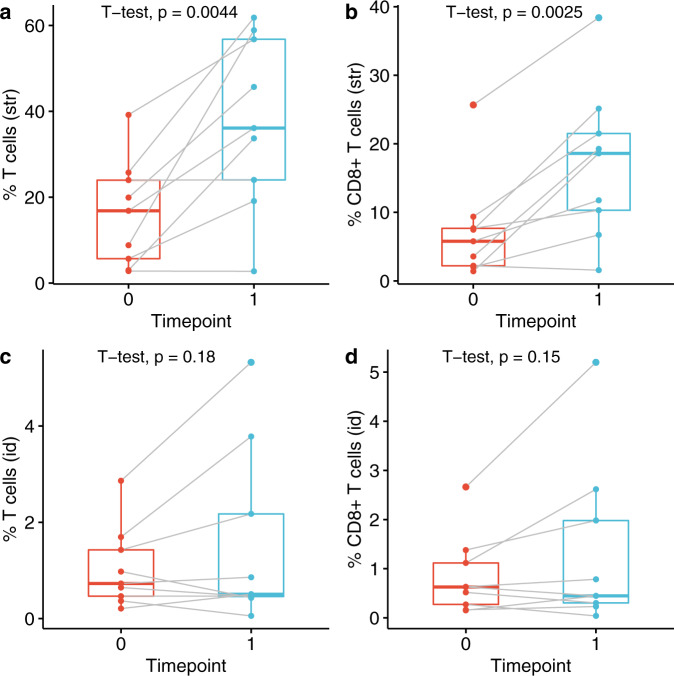
Stromal T cell densities increased in response to intralesional anti-PD-1 therapy. Total T cell (**a**, **c**) and CD8^+^ T cell (**b**, **d**) densities in the stromal regions (**a**, **b**) and intraductal regions (**c**, **d**) pre-therapy (timepoint 0) and post-therapy (timepoint 1). The bounds of each box represent the interquartile range (IQR). The horizontal line within a box is the median. Whiskers extend to the largest and smallest values no further than 1.5*IQR. Outlying points beyond the ends of the whiskers are plotted individually. Each pair of connected points represents one patient’s data; *p*-values are derived from paired *t*-tests.

We further subdivided the DCIS microenvironment into stromal and intraductal regions using a tissue segmentation algorithm (inForm 2.4 software) and analyzed T cell densities in each region separately. We found significant expansion of both total T cells and CD8^+^ T cells in the stroma but not in the intraductal regions (Fig. 4).

### Ratios of macrophages and Treg cells to T cell populations

**Fig. 5:**
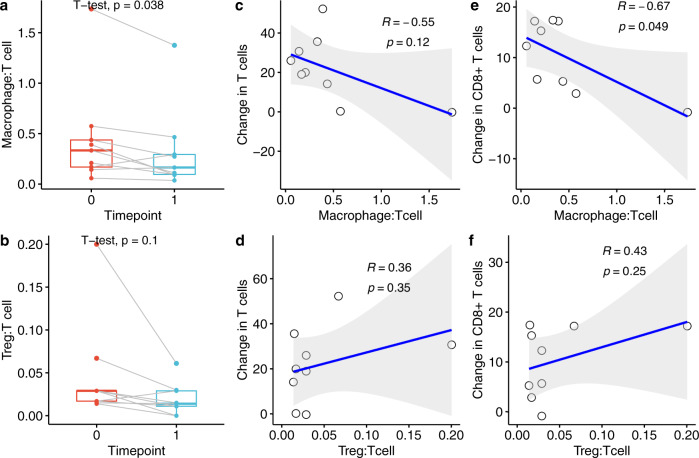
Changes in macrophage:T cell and Treg:T cell ratios in response to intralesional anti-PD-1 therapy. **a**, **b** Comparison of pre-therapy (timepoint 0) and post-therapy (timepoint 1) macrophage:T cell ratio (**a**) and Treg:T cell ratio (**b**). The bounds of each box represent the interquartile range (IQR). The horizontal line within a box is the median. Whiskers extend to the largest and smallest values no further than 1.5*IQR. Outlying points beyond the ends of the whiskers are plotted individually. Each pair of connected points represents one patient’s data; *p*-values are derived from paired *t*-tests. **c**–**f** correlations between macrophage:T cell and Treg:T cell ratios and the change, pre-therapy versus post-therapy, in T cells (**c**, **d**) and in CD8+ T cells (**e**, **f**); *R* and *p* from Pearson’s correlation. Gray shaded area represents 95% confidence region.

Macrophages and regulatory T cells (Treg) can play suppressive roles in the tumor microenvironment. We examined the ratios of macrophages to T cells and Treg to T cells in pre-therapy and post-therapy samples and observed a significant decrease in the macrophage:T cell ratios (*p* = 0.038) (Fig. 5a) but not the Treg:T cell ratios (Fig. 5b). Interestingly, the pre-therapy macrophage:T ratio inversely correlated with a change in CD8^+^ T cell densities (Fig. 5e).

### The densities of various T cell populations correlate with PD-L1^+^ tumor cell and PD-L1^+^ macrophage densities in pre-treatment samples

Staining for PD-L1 demonstrated low levels of PD-L1 positive tumor cells (CK^+^PD-L1^+^) or immune cells (PD-L1^+^ T cells and macrophages) in pre-treatment biopsies of DCIS. Three of nine (33%) cases showed PD-L1 expression in greater than 1% of tumor cells. Four cases (44%) also showed PD-L1 expression in greater than 1% of CD68^+^ macrophages or CD3^+^ T cells. Two of the cases with PD-L1^+^ tumors also showed PD-L1 expression on immune cells.

**Fig. 6:**
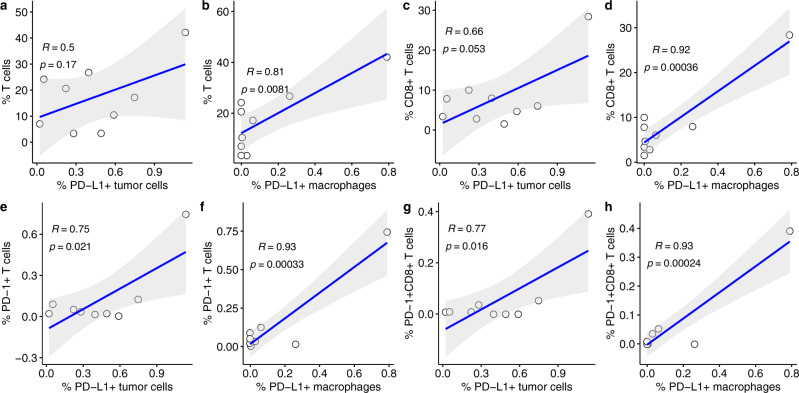
Densities of pre-treatment T cell populations correlated with pre-treatment PD-L1^+^ cell populations. Correlation plots of pre-treatment densities of total T cells (**a**, **b**), CD8^+^ T cells (**c**, **d**), PD1^+^ T cells (**e**, **f**), and PD1^+^CD8^+^ T cells (**g**, **h**) with pre-treatment densities of PD-L1^+^ macrophages (**b**, **d**, **f**, **h**) and/or PD-L1^+^ tumor cells (**a**, **c**, **e**, **g**) are shown. *R* and *p* from Pearson’s correlation. Gray shaded area represents 95% confidence region.

We observed a significant positive correlation between total T cell densities and PD-L1^+^ macrophages (*r* = 0.81, *p* = 0.0081) in the pre-treatment biopsies (Fig. 6b). There was also a positive correlation between CD8^+^ T cell densities and PD-L1^+^ macrophages (*r* = 0.92, *p* = 0.00036) in the pre-treatment biopsies (Fig. 6d). In addition, significant positive correlations were observed between PD-1^+^ T cell densities and both PD-L1^+^ tumor cells (*r* = 0.75, *p* = 0.021) (Fig. 6e) and PD-L1^+^ macrophages (*r* = 0.93, *p* = 0.00033) (Fig. 6f). Finally, there were significant positive correlations between PD-1^+^ CD8^+^ T cell densities and both PD-L1^+^ tumor cells (*r* = 0.77, *p* = 0.016) (Fig. 6g) and PD-L1^+^ macrophages (*r* = 0.93, *p* = 0.00024) (Fig. 6h).

We next evaluated whether PD-L1 expression in the pre-treatment biopsies correlated with changes in T cell or CD8^+^ T cell densities pre-therapy versus post-therapy. There were no significant associations between the change in T cell or CD8^+^ T cell densities and PD-L1^+^ tumor cell or PD-L1^+^ macrophage densities pre-treatment (data not shown).

### Intralesional anti-PD-1 does not alter the growth of DCIS lesions

**Fig. 7:**
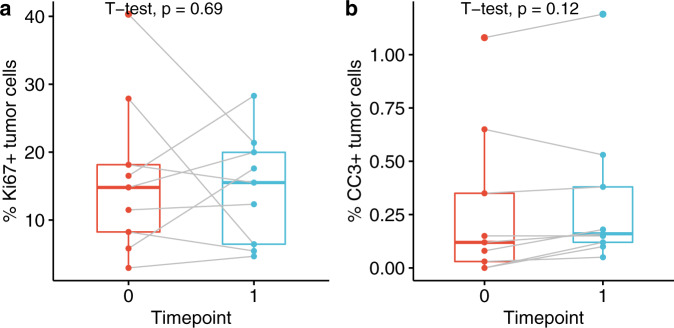
Intralesional anti-PD-1 did not decrease tumor cell proliferation or increase tumor cell apoptosis. Proliferating Ki67^+^ tumor cell densities (**a**) and cleaved caspase 3 (CC3) positive tumor cell densities (**b**) at timepoint 0 (pre-therapy) and timepoint 1 (post-therapy) are shown. The bounds of each box represent the interquartile range (IQR). The horizontal line within a box is the median. Whiskers extend to the largest and smallest values no further than 1.5*IQR. Outlying points beyond the ends of the whiskers are plotted individually. Each pair of connected points represents one patient’s data; *p*-values are derived from paired *t*-tests.

To determine if the expansion of T cells in the DCIS lesions was affecting the growth of the tumor cells, we analyzed the expression of a proliferation marker (Ki67) and a marker of apoptosis (cleaved caspase 3; CC3). As shown in Fig. 7, the densities of Ki67^+^ tumor cells and CC3^+^ tumor cells were not significantly altered by anti-PD-1 therapy when considering all patients in aggregate; however, there were two patients who did demonstrate a significant decrease in Ki67+ tumor cells in response to intralesional pembrolizumab.

### MRI volumetric assessment

Seven of nine patients underwent breast MRI exams before and following intralesional anti-PD-1 treatment. Two patients received only a baseline MRI and were, therefore, not evaluable for treatment change on MRI. DCIS volume was measured using the functional tumor volume (FTV) developed for assessment of response to neoadjuvant chemotherapy for locally advanced breast cancer^18^. Mean DCIS volume at baseline was 8.4 ml (range 0.1–48.0 ml) and after treatment was 6.4 ml (range 0.4–33.2 ml). Change in volume was negligible (within +/−1 ml) for all but one subject with the largest baseline DCIS volume of 48.0 ml, which decreased to 14.8 ml after treatment.

## Discussion

The goals of this trial were to establish the safety and feasibility of intralesional immunotherapy in patients with DCIS and to determine the response rate as defined by a significant increase in the number of T cells infiltrating the tumor immune microenvironment pre-pembrolizumab versus post-pembrolizumab therapy. This trial succeeded in achieving each of those objectives. Intralesional pembrolizumab was extremely well-tolerated with mild self-limited pain at the injection site being the only associated adverse effect. It also generated a robust total T cell and CD8+ T cell response in the tumor immune microenvironment of most patients pre-therapy versus post-therapy.

Immunotherapy delivered via mAbs targeting immune checkpoint molecules such as CTLA4, PD-1, and PD-L1 has led to a revolution in cancer therapy^19,20^. The typical route of delivery of these agents is systemic (intravenously); however, there are potential advantages of confining these agents to the tumor microenvironment by local intratumoral injection. First, the most relevant sites of action for these drugs are on the lymphocytes already infiltrating the tumor or on other cells present in the tumor microenvironment. Second, tissue penetration of systemically administered mAbs is poorly defined in solid tumors. For example, even after full doses of anti-PD-1 mAb, complete receptor occupancy of PD-1 in the tumor microenvironment is not always reached^21^. Third, given that intratumoral doses are typically much lower than systemic doses, there will be lower systemic exposure to the agents, potentially reducing systemic side effects. Finally, local administration is probably more effective at targeting the lymphoid tissue “downstream” of lymphatic drainage from the injected tumor. Several early-stage clinical trials conducted in patients with cutaneous malignancies have demonstrated the efficacy of intralesional immune-modulatory agents ^22,23^.

This study demonstrated that intralesional injection of an anti-PD-1 mAb (pembrolizumab; up to 8 mg/injection) in patients with high-risk DCIS was well-tolerated with no adverse events reported other than mild very short-term discomfort at the injection site. This is in contrast to intravenous (IV) pembrolizumab (200 mg/injection), which is associated with dermatologic toxicity in 30–40% of patients, gastrointestinal toxicity, hepatotoxicity, and endocrine toxicity, including adrenal insufficiency.

Intralesional anti-PD-1 therapy induced significant increases in total T cells and CD8+ T cells in the immune microenvironment of DCIS but not other immune cell populations (B cells, macrophages, Treg cells). Several groups have observed an increase in Ki67^+^ T cells in the peripheral blood of cancer patients receiving anti-PD-1 therapy^24–26^. In our study, we did not evaluate peripheral blood but did find that there was no significant increase in Ki67^+^ T cells within the tumor microenvironment pre-therapy versus post-therapy. This could be due to the time interval between the collection of the tissue samples (3–4 weeks). It has previously been reported that peripheral blood Ki67^+^ T cells significantly increase in the first seven days of anti-PD-1 treatment and then decrease over the next two weeks^26^.

Although we did not observe changes in macrophage densities pre-therapy versus post-therapy, we did see a significant decrease in the macrophage to T cell ratio in 8 of the 9 cases, most likely due to the increase in T cells post-therapy. The one case (pt 7) that demonstrated an increase in the macrophage to T cell ratio post-therapy had a significant increase in macrophage density (3 to 11%) compared to the increase in T cell density (17 to 36%). Interestingly, the pre-treatment macrophage: T ratios were negatively correlated with a change in CD8^+^ T cell densities, suggesting that a high pre-treatment macrophage: T cell ratio may inhibit the immunological response to anti-PD-1 therapy.

Previous studies examining PD-L1 expression in DCIS have demonstrated mixed results. In one study, none of the cases were considered DCIS tumor cell positive (defined as >5% of tumor cells being PD-L1^+^)^27^, whereas in a second study 11% of the cases were tumor cell positive (defined as >1% of tumor cells being PD-L1^+^)^28^. In our study, 33% of the cases were positive for PD-L1^+^ tumor cells (defined as >1% of tumor cells being PD-L1^+^). The use of different anti-PD-L1 antibodies in each of these studies (Thompson et al: clone 5H1; Hendry et al: clone SP263; this study: clone E1L3N), as well as the different definitions of PD-L1 tumor positivity, may explain these differences. PD-L1 was more highly expressed on immune cell infiltrates than on tumor cells in previous studies and in this study. Thompson reported 81% of the DCIS lesions contained PD-L1^+^ immune cells, Hendry reported 25% of cases with PD-L1^+^ immune cells, and we observed 44% of the DCIS cases with significant PD-L1^+^ T cells or macrophages. Immune cell PD-L1 expression is also more common than tumor cell PD-L1 expression in invasive breast cancer and is associated with high-risk histological features such as tumor grade, HER2-negativity, and hormone receptor negativity^29–31^. With respect to intralesional anti-PD-1 therapy, we observed a significant decrease in PD-L1^+^ tumor cells but not in PD-L1^+^ immune cells.

Several studies have demonstrated that DCIS lesions with associated immune infiltrate, or tumor-infiltrating lymphocytes (TILs), are also associated with high-risk features, such as young age, large size, high-grade, hormone receptor negativity, and the presence of comedonecrosis. Furthermore, DCIS with dense TILs has been found to be an independent prognostic factor, predicting a greater risk of ipsilateral recurrence and shorter recurrence-free intervals following resection^32,33^. DCIS with microinvasive carcinoma is characterized by denser TILs than pure DCIS^34^. Rather than the immune cells acting to directly enhance risk, however, they may be acting to prevent biologically aggressive DCIS lesions from acquiring invasive characteristics.

The TILs may not facilitate the development of invasive breast cancer but, instead, signify the presence of biologically aggressive premalignant disease. Dense TILs are known to be most commonly present in HER2+ and triple-negative DCIS^35^, as well as lesions with a high DCIS OncotypeDX score^36^. It has also been demonstrated that dense TILs are present in triple-negative invasive breast cancer and that these lesions respond favorably to immunotherapy^37,38^. This offers the opportunity to intervene therapeutically with immunomodulatory agents in the highest risk DCIS lesions with the goal of generating an immune response that reduces risk. These patients are at high risk of DCIS or invasive recurrence even after receiving adequate surgery. We are testing intralesional pembrolizumab to determine if it can be developed as a neoadjuvant adjunct to surgery. The goal is to improve outcomes with minimal associated risk for those who stand to benefit most. Intralesional immunotherapies have the potential to be paired with surgical resection to create a more personalized therapeutic approach for patients with high-risk DCIS.

Despite significant increases in total T cells and CD8^+^ T cells following intralesional anti-PD-1 therapy in this study, we did not observe an anti-tumor response as measured by a decrease in tumor cell proliferation, an increase in tumor cell apoptosis, or a decrease in DCIS volume kinetics measured with MRI. But there were three patients for whom some response was observed: one patient experienced a significant decrease in tumor volume, and two patients had a significant decrease in Ki67+ tumor cells.

When we subdivided the DCIS microenvironment into stromal and intraductal regions, we found significant expansion of both total T cells and CD8^+^ T cells only in the stromal region. This suggests that while intralesional anti-PD-1 elicited a robust T cell response, there may be mechanisms that prevent these cells from entering the ducts to directly interact with and kill tumor cells. It may be that more than two injections or the addition of another agent to facilitate transport across the ductal epithelium is necessary to generate a reduction of in-situ cells. It is also possible that the stromal T cells will be sufficient to prevent invasion. In this study, all patients are proceeding with surgical therapy, so it is not possible to know whether the T cell response provides some protective benefit.

In conclusion, we have demonstrated that intralesional anti-PD-1 immunotherapy for DCIS is safe and elicits a significant local expansion of T cells. We currently have a dose-expansion study underway with patients receiving 4 intralesional injections of pembrolizumab (8 mg per injection, the highest dose tested in this dose-escalation study), each spaced 3 weeks apart, and we are considering the addition of a second agent to facilitate direct T cell interaction with the DCIS tumor cells. These strategies will test whether the robust T cell infiltrate is replicable and whether a longer period of time with more sustained exposure to the agent or the addition of a second agent will result in tumor cell destruction and tumor volume reduction.

## Methods

### Patient selection

Patients with pathologically-confirmed DCIS were eligible for enrollment if they possessed at least two of the following high-risk features: young age (<45 years), large size (>5 cm), high-grade (grade II or III), a palpable mass, hormone receptor (HR) negativity, and/or HER2 positivity. When needed, p63 staining was performed to confirm a diagnosis of DCIS (Supplementary Fig. 10). This study was approved by the Institutional Review Board of the University of California, San Francisco, and all patients signed written informed consent. Patients were recruited between March 2017 and June 2018.

### Study design

This phase 1 trial (NCT 02872025) was designed as a 3 × 3 dose-escalation study that enrolled patients in three sequential cohorts. The first cohort of three patients received a dose of 2 mg intralesional pembrolizumab at two separate timepoints, each spaced three weeks apart prior to surgery. As no serious adverse events were encountered among patients in the first cohort, the second cohort of three patients was enrolled and received 4 mg intralesional pembrolizumab, again at two separate timepoints spaced three weeks apart pre-operatively. The final cohort (three patients) was then enrolled and received 8 mg intralesional pembrolizumab in the same scheme. We determined intralesional pembrolizumab dosing based upon preclinical studies of immunomodulatory antibodies administered intratumorally, which demonstrate that an intratumoral dose 1/20th to 1/100th of the typical systemic intravenous dose will generate equivalent anti-tumor effects^39–43^. These studies supported our choice of doses which were equivalent to 1/100th, 1/50th, and 1/25th of the standard intravenous dose (200 mg).

Intralesional pembrolizumab was administered at the UCSF infusion center by a breast oncology surgeon, and patients were monitored for an hour after each administration. Tissue was obtained pre-treatment from core biopsies and post-treatment from surgical resection specimens, either mastectomy or lumpectomy as determined by the treating surgeon.

The primary outcome was the determination of the maximum tolerated dose of intralesional pembrolizumab while characterizing associated toxicities and assessing the feasibility of this novel route of administration. Secondary outcomes included characterization of changes in the tumor immune microenvironment as assessed by multiplex immunofluorescence (mIF), change in tumor cell apoptosis (cleaved caspase 3 (CC3) staining), change in tumor cell proliferation (Ki67 staining), and change in DCIS volume kinetics by MRI following intralesional pembrolizumab treatment.

A control group was not selected for this phase I pilot study, as this study was primarily designed to evaluate the safety and efficacy of intralesional pembrolizumab, as well as any changes that occurred in the tumor immune microenvironment.

### Multiplex immunofluorescence staining and analysis

mIF was performed on 4 μm sections obtained from FFPE tumor blocks using the Opal 7-color IHC kit (Akoya Biosciences, Marlborough, MA). Staining was performed on a Ventana Discovery Ultra autostainer (Ventana/Roche, Indianapolis, IN) with antibodies against the following: pan-cytokeratin AE1/AE3 (epithelial cells, 1:200 dilution, cat# M3515, Dako, Carpinteria, CA), CD3 (T cells, clone 2GV6, ready to use, cat# 790–4341, Ventana/Roche), CD8 (cytotoxic T cells, clone 4B11, 1:100 dilution, cat# CD8–4B11-L-CE, Leica Biosystems, Buffalo Grove, IL), CD20 (B cells, clone L26, ready to use, cat# 790–2531, Ventana/Roche), CD68 (macrophages, clone PG-M1, 1:100 dilution, cat# M0876, Dako), Foxp3 (regulatory T cells, clone SP97, 1:25 dilution, cat# M3972, Spring Bioscience, Pleasanton, CA), PD-1 (clone EPR4877, 1:100 dilution, cat# ab137132, abcam, Cambridge, MA), PD-L1 (clone E1L3N, 1:100 dilution, cat# 1364e, Cell Signaling Technology, Danvers, MA), and Ki67 (proliferation marker, clone 30–9, ready to use, cat# 790–4286, Ventana/Roche). Each section was run through sequential rounds of heat-inactivation epitope retrieval (HIER), followed by incubation with primary antibody, then secondary horseradish peroxidase (HRP)-conjugated antibody. The primary antibody was visualized using different tyramide-linked Opal fluorophores (Akoya Biosciences). Subsequent HIER steps removed bound antibodies before applying the next round of primary antibody, secondary HRP conjugate, and tyramide-Opal dye. After the final antibody sequence, sections were counterstained with DAPI (Akoya Biosciences) and mounted with Vectashield fluorescence mounting medium (Vector Labs, Burlingame, CA). The sequence of antibody labeling was determined based on the target antigen’s stability to repeated HIER steps. Each primary antibody was matched with an appropriate Opal TSA dye based on staining intensity and on the antigen’s cellular localization to minimize potential signal crossover of co-localized targets.

The mIF stained slides were scanned with a Vectra 3.0 automated quantitative pathology imaging system (Akoya Biosciences). Fluorescent spectra from 420 nm to 720 nm were captured from each slide at 20-nm intervals, and the resulting images were combined to create a single stack image that retained the particular spectral signature of all IF markers^44^. Sections of a tonsil specimen were stained as a control to calibrate the spectral image protocol. Whole slide scans were first obtained at low magnification (×4), then 15–20 regions of interest (ROIs) per case were randomly selected using Phenochart (Akoya Biosciences) and were scanned at high resolution (×20) for subsequent multispectral unmixing and analysis.

A spectral library containing the emitting spectral peaks of all fluorophores was created with inForm 2.4 image analysis software (Akoya Biosciences) using multispectral images obtained from single stained slides for each fluorophore. This spectral library was then used to separate each multispectral image into its individual components (spectral unmixing). Tissue and cell segmentation were performed using inForm 2.4 algorithms. Spectrally unmixed and segmented images were then subjected to a machine learning cell phenotyping algorithm in inForm 2.4. Cell populations included total T cells (CD3^+^), cytotoxic T cells (CD3^+^CD8^+^), Treg cells (CD3^+^Foxp3^+^), B cells (CD20^+^), macrophages (CD68^+^), and tumor cells (CK^+^). Densities of immune cell populations were calculated as a percentage of total cells. Densities of PD-1^+^ or PD-L1^+^ cells were reported as a percentage of the given cell population. All phenotyping and quantification were performed blinded to patient identity and clinical outcomes.

### Statistical analysis

Comparisons between cell populations pre-therapy and post-therapy were analyzed using paired, two-tailed Student’s *t*-test statistical analyses in R version 3.6.0. Correlations between various cell populations were analyzed using Pearson’s correlation, also performed using R 3.6.0 software.

### Reporting summary

Further information on research design is available in the Nature Research Reporting Summary linked to this article.

## Supplementary information

Supplementary Information

Reporting Summary

## Data Availability

The data generated and analyzed during this study are described in the following data record: 10.6084/m9.figshare.14125391^45^. The data are openly available as part of the data record in the file ‘Immune Infiltrates Data.xls’. This spreadsheet contains 6 tabs labeled according to the figure it underlies.

## Code availability

The R code used to perform statistical analyses comparing cell populations pre-therapy and post-therapy in this study is available from the corresponding author upon reasonable request.

## References

[1] Narod SA, Iqbal J, Giannakeas V, Sopik V, Sun P (2015). Breast cancer mortality after a diagnosis of ductal carcinoma in situ. JAMA Oncol..

[2] Campbell MJ (2017). Characterizing the immune microenvironment in high-risk ductal carcinoma in situ of the breast. Breast Cancer Res. Treat..

[3] Hanahan D, Weinberg RA (2011). Hallmarks of cancer: the next generation. Cell.

[4] Gabrilovich DI, Nagaraj S (2009). Myeloid-derived suppressor cells as regulators of the immune system. Nat. Rev. Immunol..

[5] Whiteside TL (2014). Induced regulatory T cells in inhibitory microenvironments created by cancer. Expert Opin. Biol. Ther..

[6] Campbell MJ (2011). Proliferating macrophages associated with high grade, hormone receptor negative breast cancer and poor clinical outcome. Breast Cancer Res. Treat..

[7] Mukhtar RA (2012). Elevated levels of proliferating and recently migrated tumor associated macrophages confer increased aggressiveness and worse outcomes in breast cancer. Ann. Surg. Oncol..

[8] Obeid E, Nanda R, Fu YX, Olopade OI (2013). The role of tumor-associated macrophages in breast Cancer progression. Int. J. Oncol..

[9] de la Cruz-Merino, L. et al. New insights into the role of the immune microenvironment in breast carcinoma. *Clin. Dev. Immunol*. 10.1155/2013/785317. (2013). 10.1155/2013/785317 PMC3686058 23861693

[10] Campbell MJ (2013). The prognostic implications of macrophages expressing proliferating cell nuclear antigen in breast cancer depend on immune context. PLoS ONE.

[11] Hussein MR, Hassan HI (2006). Analysis of the mononuclear inflammatory cell infiltrate in the normal breast, benign proliferative breast disease, in situ and infiltrating ductal breast carcinomas: preliminary observations. J. Clin. Pathol..

[12] Esserman LJ (2006). Magnetic resonance imaging captures the biology of ductal carcinoma in situ. J. Clin. Oncol..

[13] Sharma M (2010). Analysis of stromal signatures in the tumor microenvironment of ductal carcinoma in situ. Breast Cancer Res. Treat..

[14] Greenwald RJ, Freeman GJ, Sharpe AH (2005). The B7 family revisited. Annu Rev. Immunol..

[15] Okazaki T, Maeda A, Nishimura H, Kurosaki T, Honjo T (2001). PD-1 immunoreceptor inhibits B cell receptor mediated signaling by recruiting src homology 2-domain-containing tyrosine phosphatase 2 to phosphotyrosine. Proc. Natl Acad. Sci. USA.

[16] Nanda R (2017). Pembrolizumab plus standard neoadjuvant therapy for high-risk breast cancer (BC): Results from I-SPY 2. J. Clin. Oncol..

[17] Nanda R (2020). Effect of Pembrolizumab Plus neoadjuvant chemotherapy on pathologic complete response in women with early-stage breast cancer: An analysis of the ongoing Phase 2 adaptively randomized I-SPY2 trial. JAMA Oncol..

[18] Hylton NM (2012). Locally advanced breast cancer: MR imaging for prediction of response to neoadjuvant chemotherapy—results from ACRIN 6657/I-SPY TRIAL. Radiology.

[19] Sharma P, Allison JP (2015). The future of immune checkpoint therapy. Science.

[20] Topalian SL, Drake CG, Pardoll DM (2015). Immune checkpoint blockade: a common denominator approach to cancer therapy. Cancer Cell..

[21] Das R (2015). Combination therapy with anti-CTLA-4 and anti-PD-1 leads to distinct immunologic changes in vivo. J. Immunol..

[22] Hofmann M (2008). Phase 1 evaluation of intralesionally injected TLR9-agonist PF-3512676 in patients with basal cell carcinoma or metastatic melanoma. J. Immunother..

[23] Nouri N, Garbe C (2016). Intralesional immunotherapy as a strategy to treat melanoma. Expert Opin. Biol. Ther..

[24] Kamphorst AO (2017). Proliferation of PD-1 + CD8 T cells in peripheral blood after PD-1-targeted therapy in lung cancer patients. Proc. Natl Acad. Sci. USA.

[25] Huang A (2017). T-cell invigoration to tumour burden ratio associated with anti-PD-1 response. Nature.

[26] Kim KH (2019). The first-week proliferative response of peripheral blood PD-1^+^ CD8^+^ T cells predicts the response to anti-PD-1 therapy in solid tumors. Clin. Cancer Res..

[27] Thompson E (2016). The immune microenvironment of breast ductal carcinoma in situ. Mod. Pathol..

[28] Hendry S (2017). Relationship of the breast ductal carcinoma in situ immune microenvironment with clinicopathological and genetic features. Clin. Cancer Res..

[29] Mittendorf EA (2014). PD-L1 expression in triple-negative breast cancer. Cancer Immunol. Res..

[30] Cimino-Mathews A (2016). PD-L1 (B7-H1) expression and the immune tumor microenvironment in primary and metastatic breast carcinomas. Hum. Pathol..

[31] Ghebeh H (2006). The B7-H1 (PD-L1) T lymphocyte-inhibitory molecule is expressed in breast cancer patients with infiltrating ductal carcinoma: correlation with important high-risk prognostic factors. Neoplasia.

[32] Darvishian F (2019). Tumor-infiltrating lymphocytes in a contemporary cohort of women with ductal carcinoma in situ (DCIS). Ann. Aurg. Oncol..

[33] Toss MS (2018). Prognostic significance of tumor-infiltrating lymphocytes in ductal carcinoma in situ of the breast. Mod. Pathol..

[34] Beguinot M (2018). Analysis of tumour-infiltrating lymphocytes reveals two new biologically different subgroups of breast ductal carcinoma in situ. BMC Cancer.

[35] Agahozo MC, Hammerl D, Debets R, Kok M, van Deurzen CHM (2018). Tumor-infiltrating lymphocytes and ductal carcinoma in situ of the breast: friends or foes?. Mod. Pathol..

[36] Knopfelmacher A, Fox J, Lo Y, Shapiro N, Fineberg S (2015). Correlation of histopathologic features of ductal carcinoma in situ of the breast with the oncotype DX DCIS score. Mod. Pathol..

[37] Schmid P (2020). Pembrolizumab for early triple-negative breast cancer. NEJM.

[38] Nanda, R. et al. Effect of pembrolizumab plus neoadjuvant chemotherapy on pathologic complete response in women with early-stage breast cancer. *JAMA Oncol*. **6**, 676–684 (2020). 10.1001/jamaoncol.2019.6650 PMC7058271 32053137

[39] Marabelle A (2013). Depleting tumor-specific Tregs at a single site eradicates disseminated tumors. J. Clin. Invest..

[40] Palazón A (2012). The HIF-1α hypoxia response in tumor-infiltrating T lymphocytes induces functional CD137 (4–1BB) for immunotherapy. Cancer Discov..

[41] Van de Voort TJ, Felder MA, Yang RK, Sondel PM, Rakhmilevich AL (2013). Intratumoral delivery of low doses of anti-CD40 mAb combined with monophosphoryl lipid a induces local and systemic antitumor effects in immunocompetent and T cell-deficient mice. J. Immunother..

[42] Sandin LC (2014). Local CTLA4 blockade effectively restraings experimental pancreatic adenocarcinoma growth in vivo. Oncoimmunology.

[43] Dai M, Yip YY, Hellstrom I, Hellstrom KE (2015). Curing mice with large tumors by locally delivering combinations of immunomodulatory antibodies. Clin. Cancer Res..

[44] Stack EC, Wang C, Roman KA, Hoyt CC (2014). Multiplexed immunohistochemistry, imaging, and quantitation: a review, with an assessment of Tyramide signal amplification, multispectral imaging and multiplex analysis. Methods.

[45] Glencer, A. C. et al. Metadata record for the manuscript: modulation of the immune microenvironment of high risk breast ductal carcinoma in situ by intralesional injection of pembrolizumab. *figshare*10.6084/m9.figshare.14125391 (2021). 10.1038/s41523-021-00267-z PMC8149838 34035311

