# Supplementary material for: Modulation of the immune microenvironment of high-risk ductal carcinoma in situ by intralesional pembrolizumab injection
Source: NPJ Breast Cancer. 2021 May 25;7:59. doi: 10.1038/s41523-021-00267-z (PMC8149838; doi:10.1038/s41523-021-00267-z)
Supplement: Supplementary file 1 — Supplementary Information [file 41523_2021_267_MOESM1_ESM.pdf]

Supplementary information

Modulation of the immune microenvironment of high-risk ductal carcinoma in situ by intralesional pembrolizumab injection

Alexa C. Glencer, Jasmine M. Wong, Nola M. Hylton, Gregor Krings, Emma McCune, Harriet T. Rothschild, Tristan A. Loveday, Michael D. Alvarado, Laura J. Esserman, and Michael J. Campbell

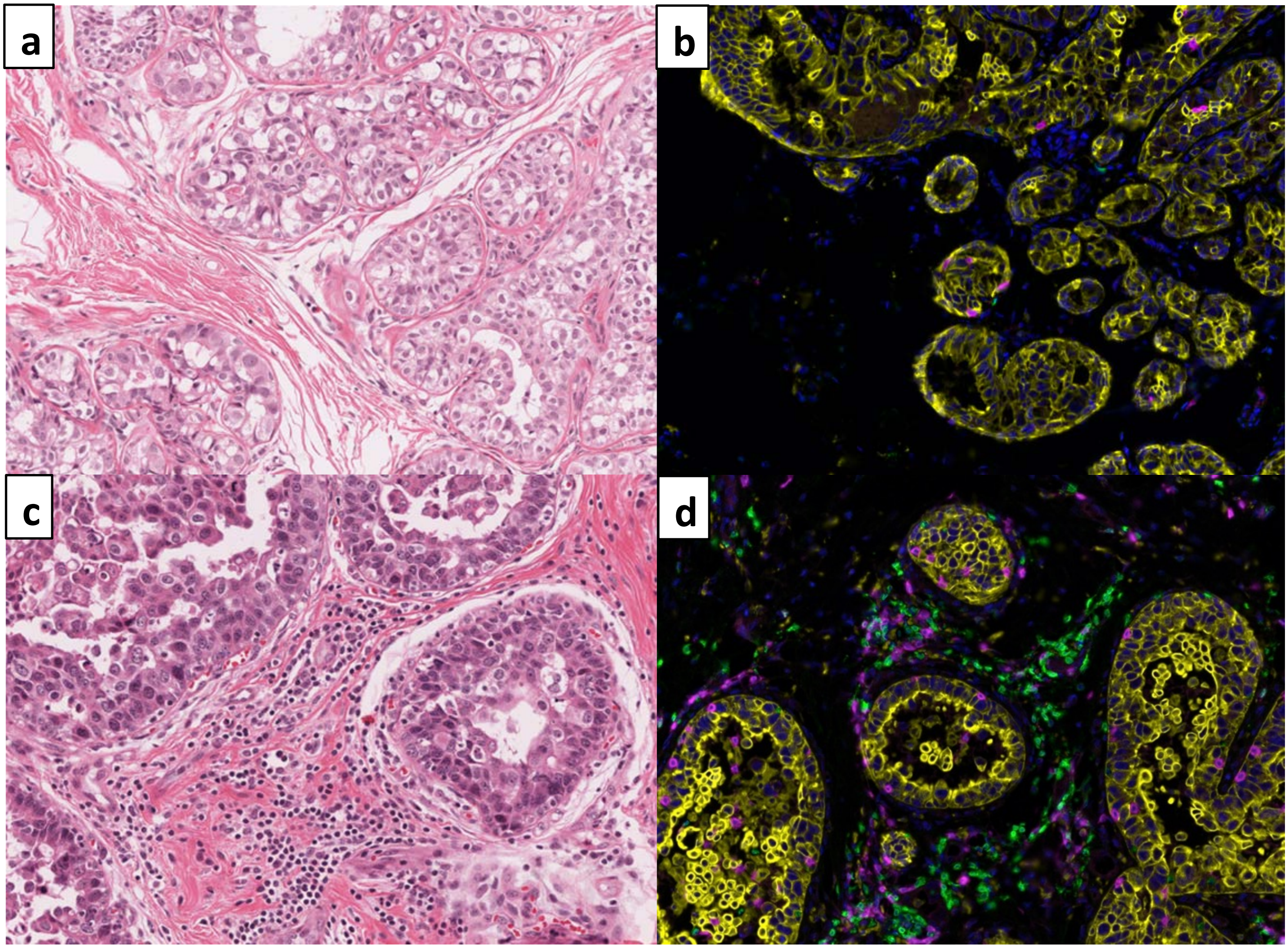

Supplementary Figure 1. T cell infiltrates prior to and following intralesional injection of pembrolizumab in patient #1. Representative hematoxylin & eosin (a, c) and multiplex immunofluorescence (mIF) images (b, d) from pre-therapy biopsies (a, b) and post-therapy surgical specimens (c, d) are shown. mIF markers: pan-cytokeratins (yellow), CD3 (green), CD8 (magenta). Original magnification 20X.

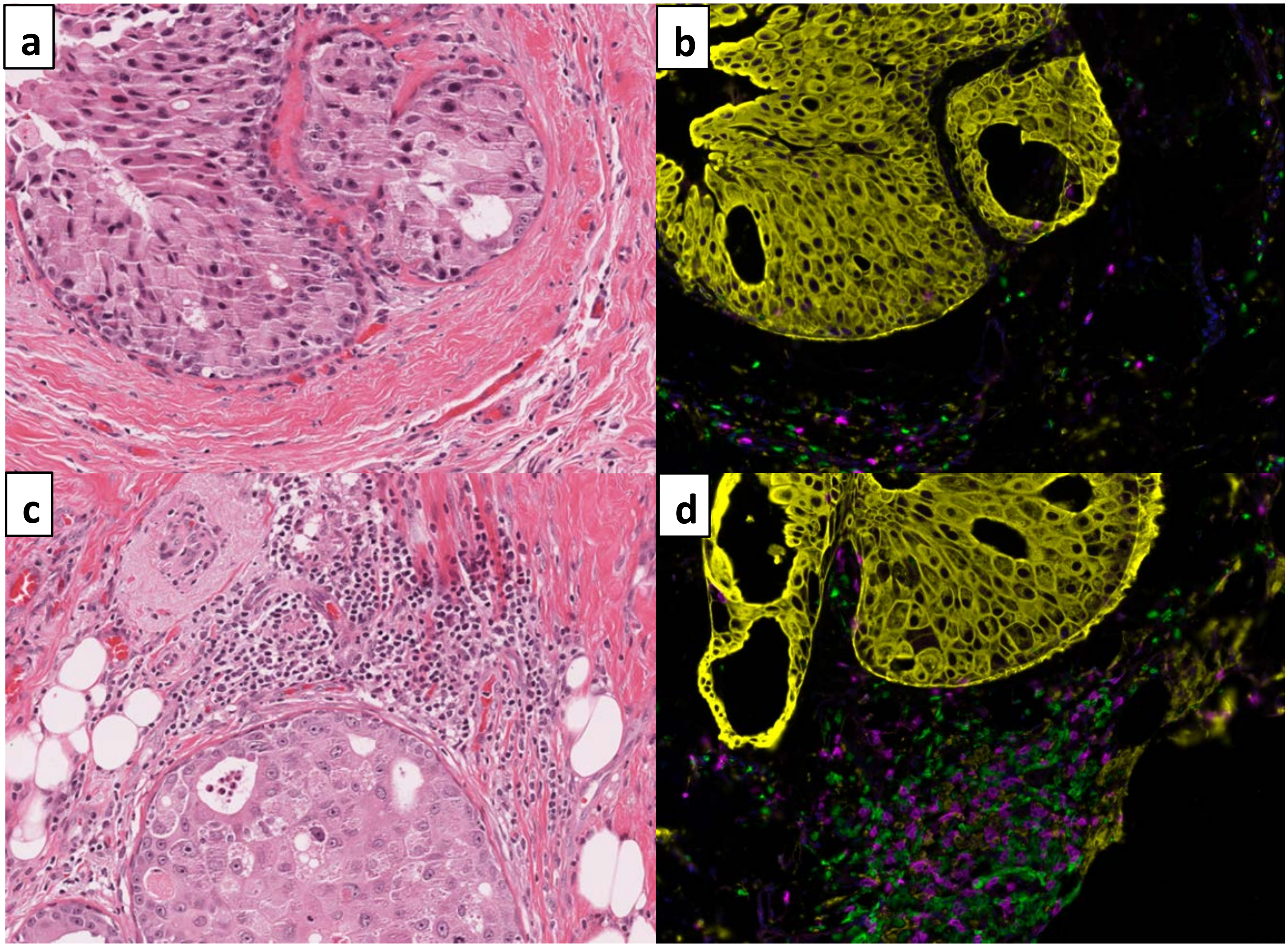

Supplementary Figure 2. T cell infiltrates prior to and following intralesional injection of pembrolizumab in patient #2. Representative hematoxylin & eosin (a, c) and multiplex immunofluorescence (mIF) images (b, d) from pre-therapy biopsies (a, b) and post-therapy surgical specimens (c, d) are shown. mIF markers: pan-cytokeratins (yellow), CD3 (green), CD8 (magenta). Original magnification 20X.

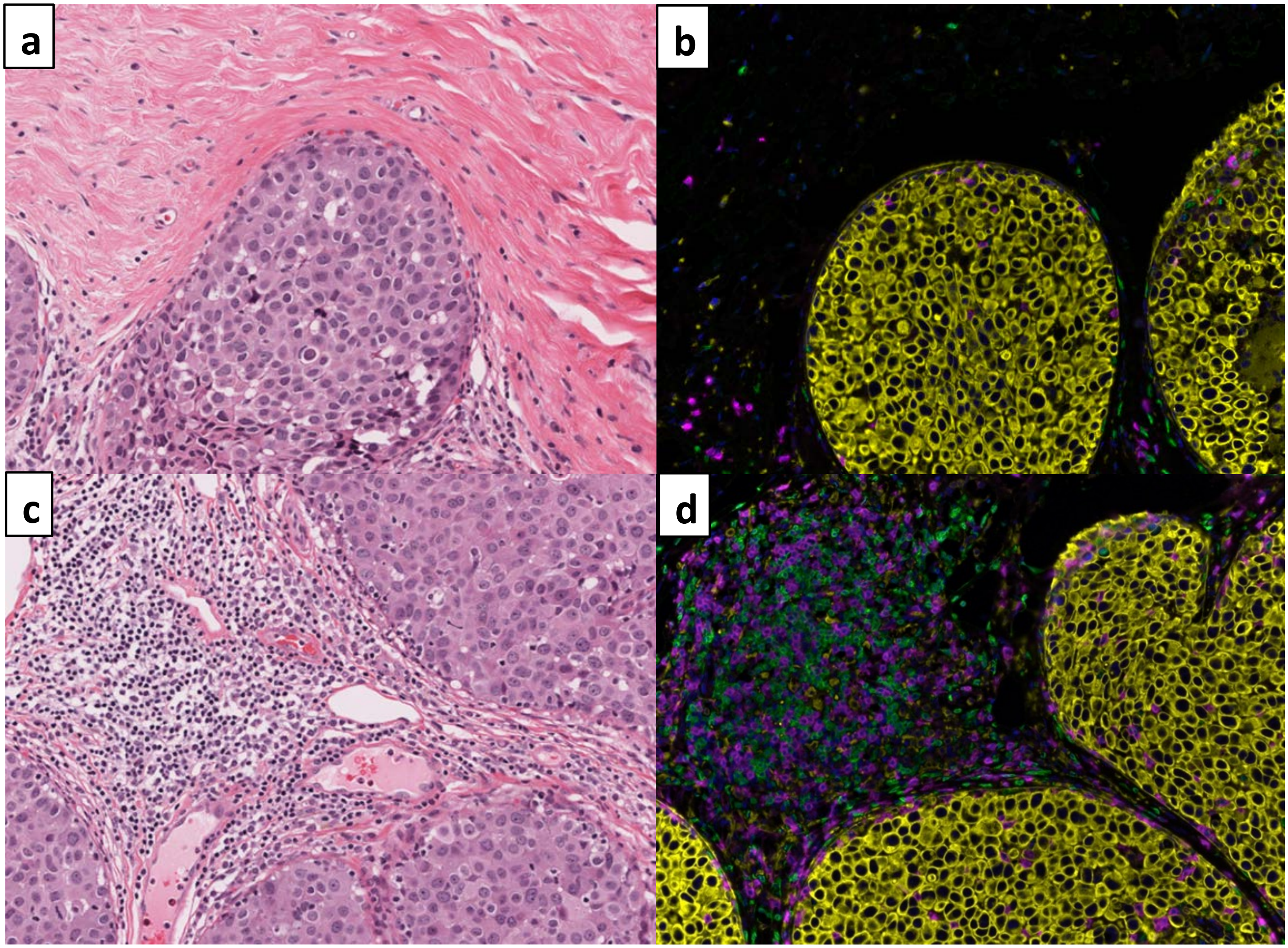

Supplementary Figure 3. T cell infiltrates prior to and following intralesional injection of pembrolizumab in patient #3. Representative hematoxylin & eosin (a, c) and multiplex immunofluorescence (mIF) images (b, d) from pre-therapy biopsies (a, b) and post-therapy surgical specimens (c, d) are shown. mIF markers: pan-cytokeratins (yellow), CD3 (green), CD8 (magenta). Original magnification 20X.

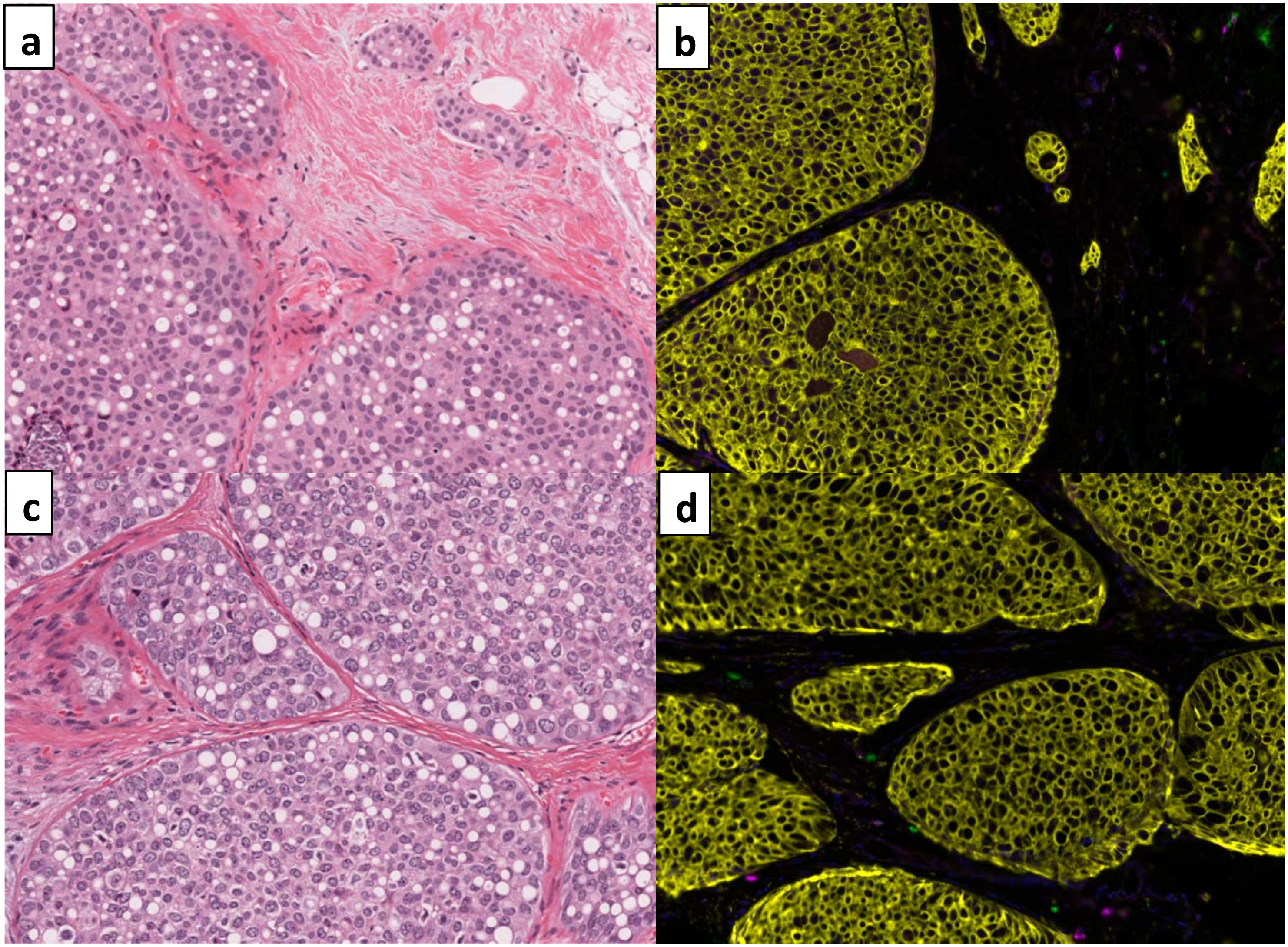

Supplementary Figure 4. T cell infiltrates prior to and following intralesional injection of pembrolizumab in patient #4. Representative hematoxylin & eosin (a, c) and multiplex immunofluorescence (mIF) images (b, d) from pre-therapy biopsies (a, b) and post-therapy surgical specimens (c, d) are shown. mIF markers: pan-cytokeratins (yellow), CD3 (green), CD8 (magenta). Original magnification 20X.

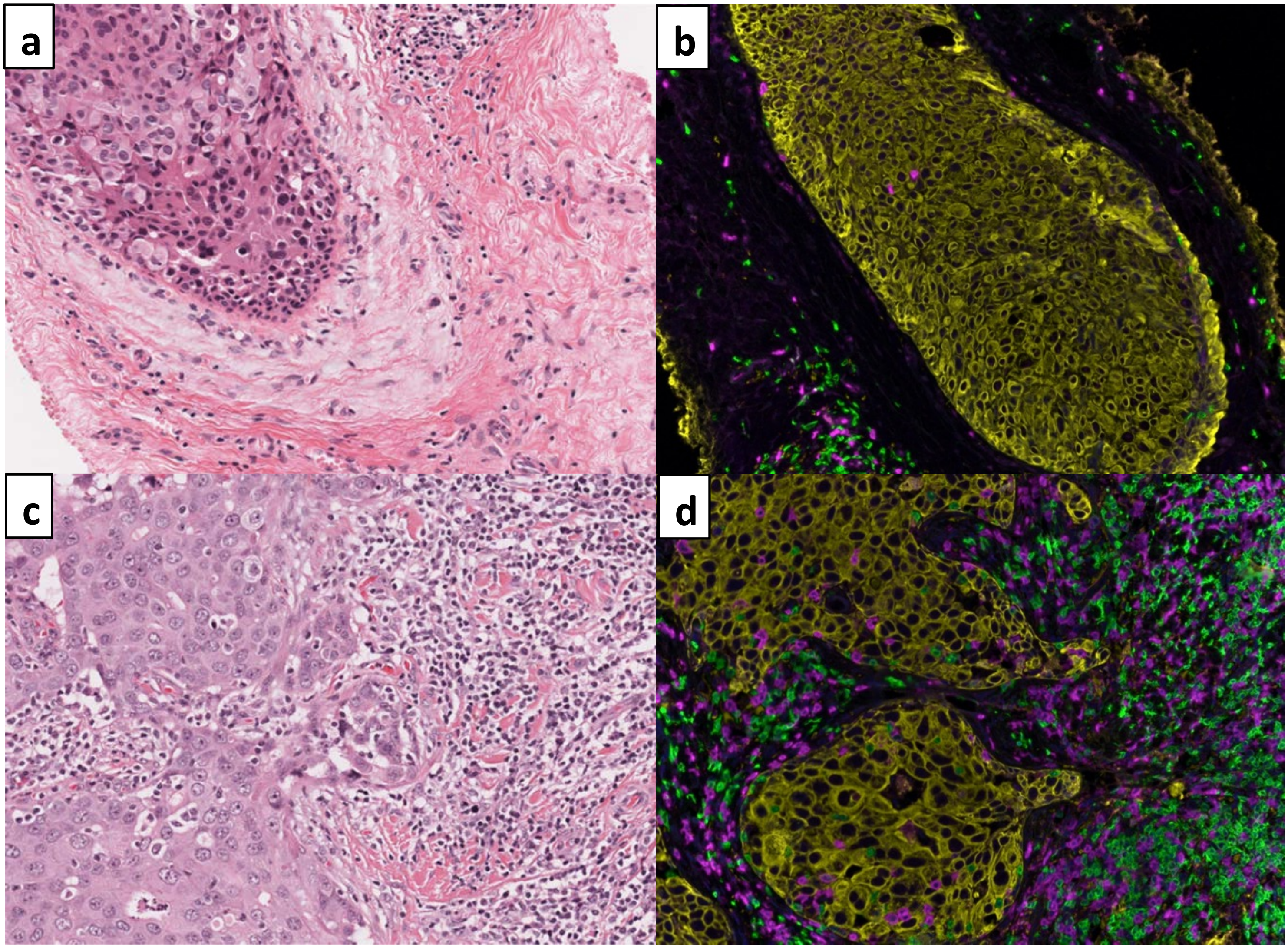

Supplementary Figure 5. T cell infiltrates prior to and following intralesional injection of pembrolizumab in patient #5. Representative hematoxylin & eosin (a, c) and multiplex immunofluorescence (mIF) images (b, d) from pre-therapy biopsies (a, b) and post-therapy surgical specimens (c, d) are shown. mIF markers: pan-cytokeratins (yellow), CD3 (green), CD8 (magenta). Original magnification 20X.

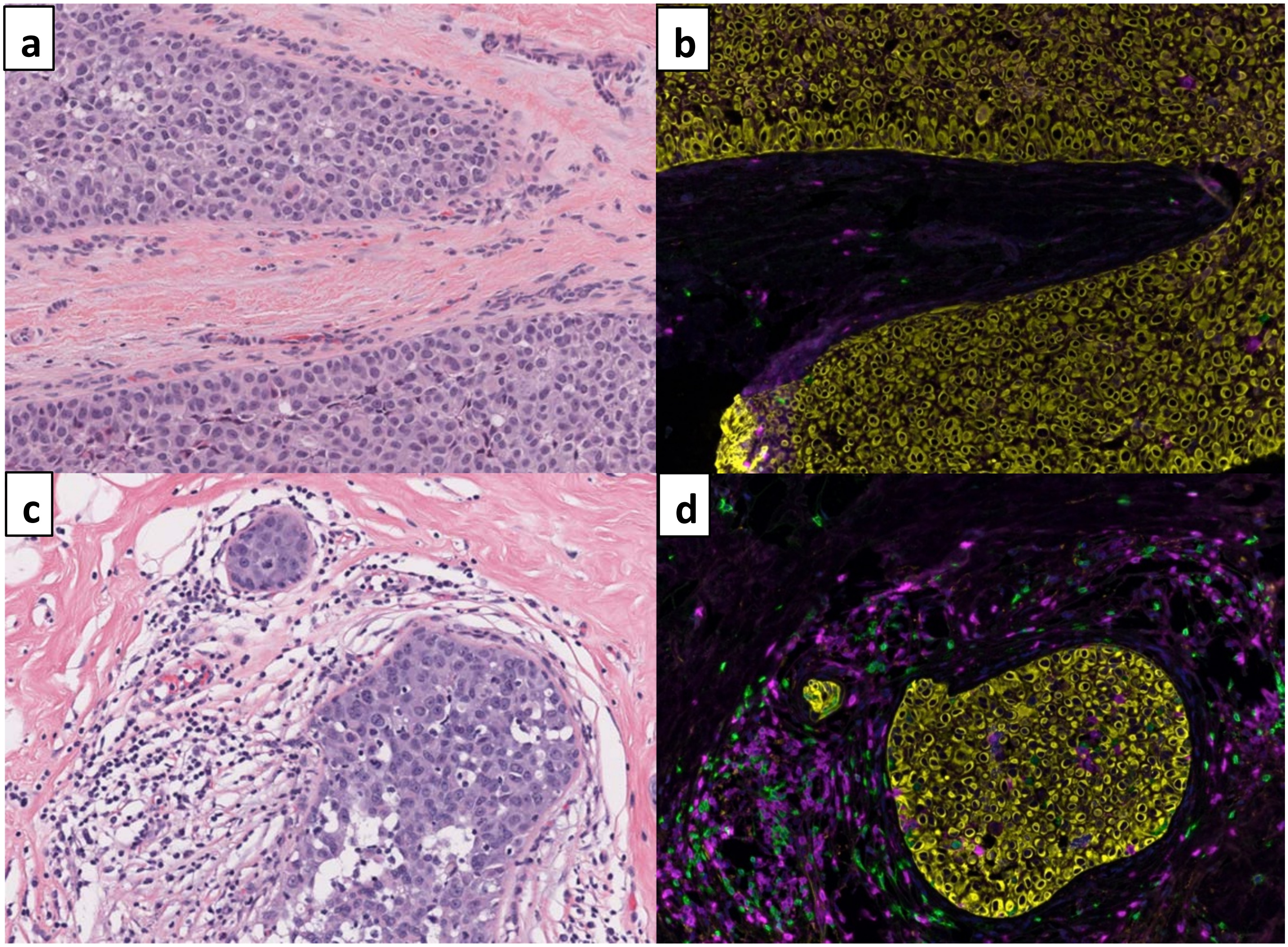

Supplementary Figure 6. T cell infiltrates prior to and following intralesional injection of pembrolizumab in patient #6. Representative hematoxylin & eosin (a, c) and multiplex immunofluorescence (mIF) images (b, d) from pre-therapy biopsies (a, b) and post-therapy surgical specimens (c, d) are shown. mIF markers: pan-cytokeratins (yellow), CD3 (green), CD8 (magenta). Original magnification 20X.

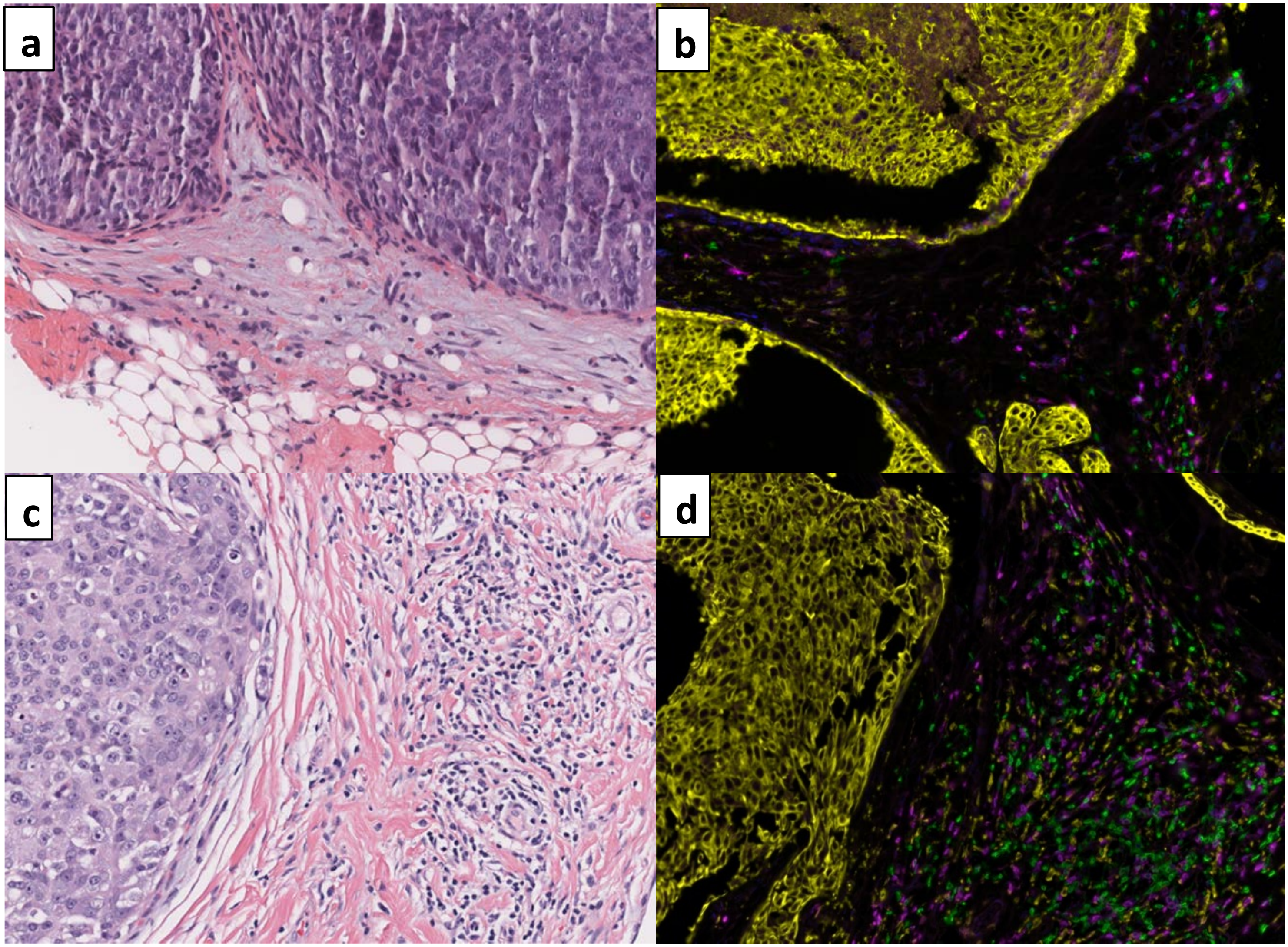

Supplementary Figure 7. T cell infiltrates prior to and following intralesional injection of pembrolizumab in patient #7. Representative hematoxylin & eosin (a, c) and multiplex immunofluorescence (mIF) images (b, d) from pre-therapy biopsies (a, b) and post-therapy surgical specimens (c, d) are shown. mIF markers: pan-cytokeratins (yellow), CD3 (green), CD8 (magenta). Original magnification 20X.

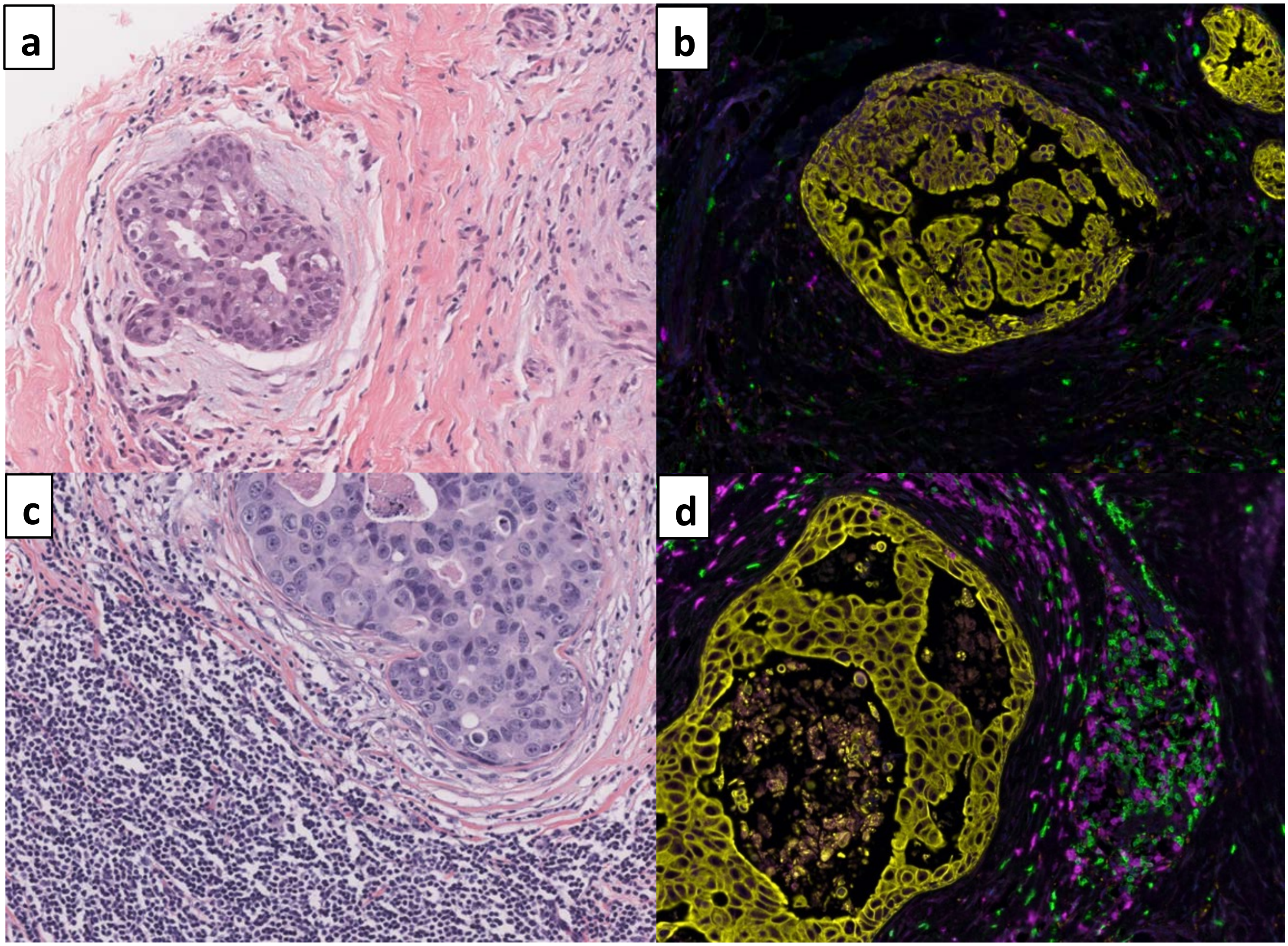

Supplementary Figure 8. T cell infiltrates prior to and following intralesional injection of pembrolizumab in patient #8. Representative hematoxylin & eosin (a, c) and multiplex immunofluorescence (mIF) images (b, d) from pre-therapy biopsies (a, b) and post-therapy surgical specimens (c, d) are shown. mIF markers: pan-cytokeratins (yellow), CD3 (green), CD8 (magenta). Original magnification 20X.

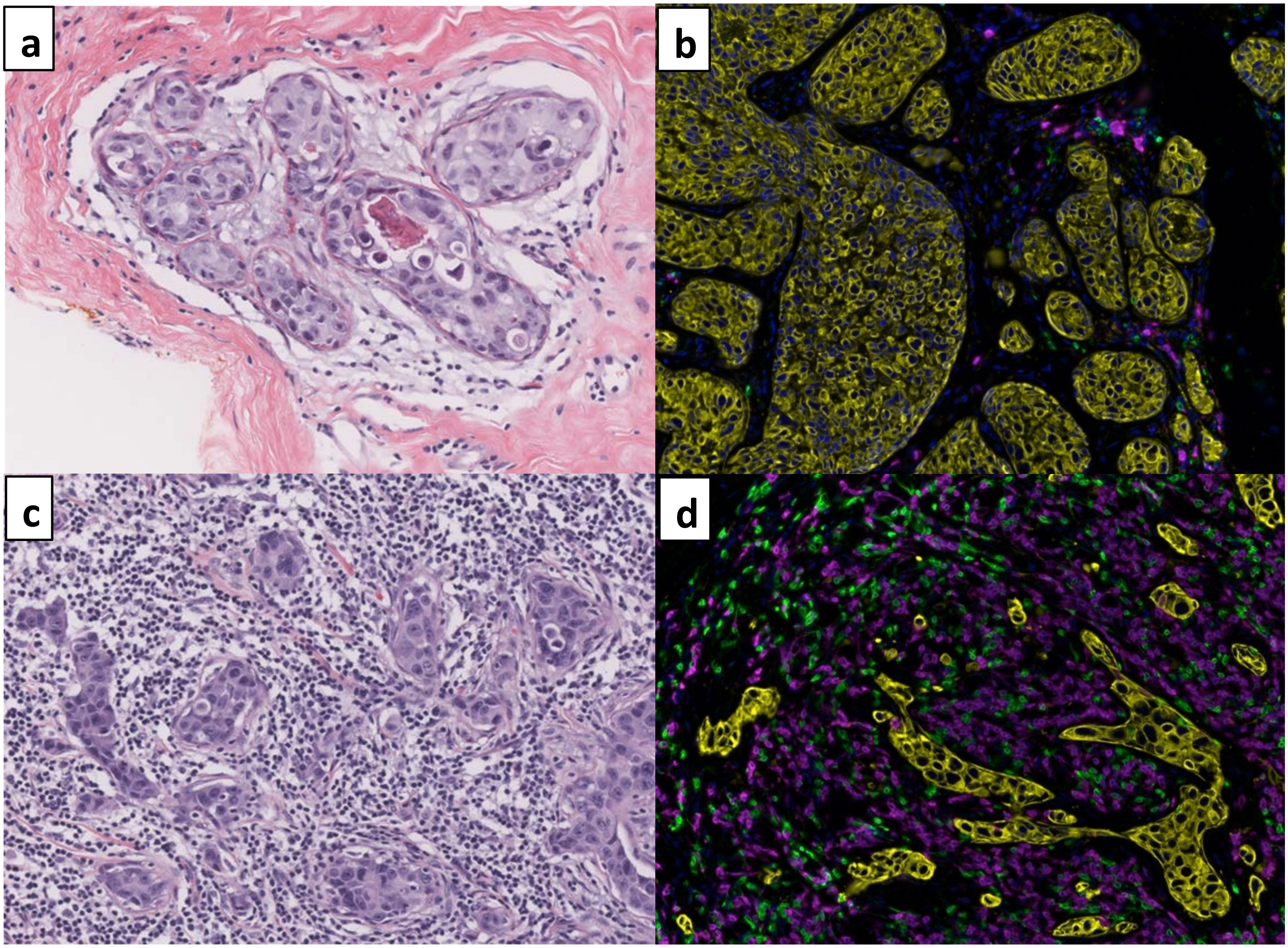

Supplementary Figure 9. T cell infiltrates prior to and following intralesional injection of pembrolizumab in patient #9. Representative hematoxylin & eosin (a, c) and multiplex immunofluorescence (mIF) images (b, d) from pre-therapy biopsies (a, b) and post-therapy surgical specimens (c, d) are shown. mIF markers: pan-cytokeratins (yellow), CD3 (green), CD8 (magenta). Original magnification 20X.

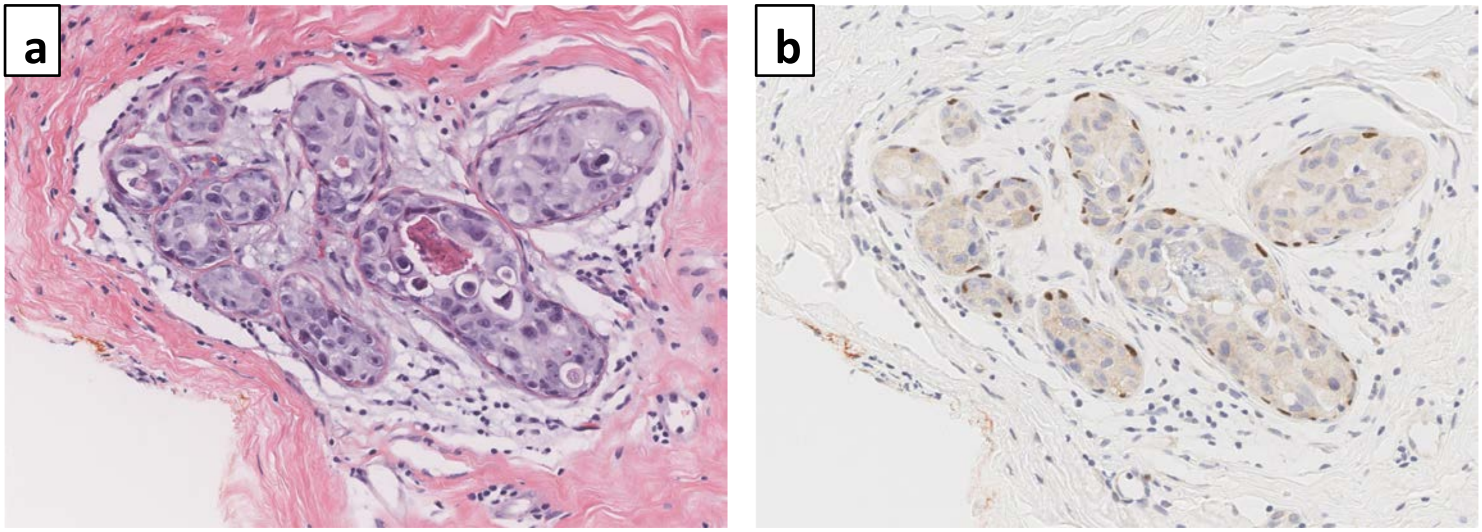

Supplementary Figure 10. Hematoxylin & eosin (a) and p63 staining (b) from pre-therapy biopsy of Pt9. Original magnification 20X.
