# Supplementary material for: Modulation of the immune microenvironment of high-risk ductal carcinoma in situ by intralesional pembrolizumab injection
Source: NPJ Breast Cancer. 2021 May 25;7:59. doi: 10.1038/s41523-021-00267-z (PMC8149838; doi:10.1038/s41523-021-00267-z)
Supplement: Supplementary file 2 — Reporting Summary [file 41523_2021_267_MOESM2_ESM.pdf]

nature research

Corresponding author(s): Michael Campbell, PhD

Last updated by author(s): 02/13/2021

Reporting Summary

Nature Research wishes to improve the reproducibility of the work that we publish. This form provides structure for consistency and transparency in reporting. For further information on Nature Research policies, see our Editorial Policies and the Editorial Policy Checklist.

Statistics

For all statistical analyses, confirm that the following items are present in the figure legend, table legend, main text, or Methods section.

n/a	Confirmed	
☐	☒	The exact sample size (n) for each experimental group/condition, given as a discrete number and unit of measurement
☐	☒	A statement on whether measurements were taken from distinct samples or whether the same sample was measured repeatedly
☐	☒	The statistical test(s) used AND whether they are one- or two-sided<br>*Only common tests should be described solely by name; describe more complex techniques in the Methods section.*
☐	☒	A description of all covariates tested
☐	☒	A description of any assumptions or corrections, such as tests of normality and adjustment for multiple comparisons
☐	☒	A full description of the statistical parameters including central tendency (e.g. means) or other basic estimates (e.g. regression coefficient) AND variation (e.g. standard deviation) or associated estimates of uncertainty (e.g. confidence intervals)
☐	☒	For null hypothesis testing, the test statistic (e.g. F, t, r) with confidence intervals, effect sizes, degrees of freedom and P value noted<br>*Give P values as exact values whenever suitable.*
☒	☐	For Bayesian analysis, information on the choice of priors and Markov chain Monte Carlo settings
☒	☐	For hierarchical and complex designs, identification of the appropriate level for tests and full reporting of outcomes
☐	☒	Estimates of effect sizes (e.g. Cohen's d, Pearson's r), indicating how they were calculated

Our web collection on statistics for biologists contains articles on many of the points above.

Software and code

Policy information about availability of computer code

Data collection	Multiplex immunofluorescence (mIF) slides were scanned using a Vectra 3.0 system (PerkinElmer), and regions of interest were selected using PhenoChart (PerkinElmer). A spectral library was generated using inForm 2.4 image analysis software (PerkinElmer). Tissue and cell segmentation were performed using inForm 2.4 algorithms (PerkinElmer).
Data analysis	Spectrally unmixed and segmented images were subjected to a machine learning cell phenotyping algorithm in inForm 2.4 (PerkinElmer). Statistical anlayses comparing cell populations were performed using R version 3.6.0 software.

For manuscripts utilizing custom algorithms or software that are central to the research but not yet described in published literature, software must be made available to editors and reviewers. We strongly encourage code deposition in a community repository (e.g. GitHub). See the Nature Research guidelines for submitting code & software for further information.

Data

Policy information about availability of data

All manuscripts must include a data availability statement. This statement should provide the following information, where applicable:

- Accession codes, unique identifiers, or web links for publicly available datasets
- A list of figures that have associated raw data
- A description of any restrictions on data availability

The datasets generated during and/or analyzed during the current study are available from the corresponding author upon reasonable request.

Field-specific reporting

Please select the one below that is the best fit for your research. If you are not sure, read the appropriate sections before making your selection.

☒ Life sciences ☐ Behavioural & social sciences ☐ Ecological, evolutionary & environmental sciences

For a reference copy of the document with all sections, see nature.com/documents/nr-reporting-summary-flat.pdf

Life sciences study design

All studies must disclose on these points even when the disclosure is negative.

Sample size	Our sample size of n = 9 was selected based upon the 3x3 dose-escalation schema of this phase I clinical trial.
Data exclusions	No data was excluded from our analysis.
Replication	This manuscript reports results from a phase I dose-escalation study for which replication is not a relevant metric.
Randomization	This was not a randomized clinical trial. This phase I study was designed to evaluate the safety and efficacy of intralesional pembrolizumab administered to high-risk breast ductal carcinoma in situ (DCIS) patients, as well as to evaluate changes in the tumor immune microenvironment in response to therapy. In our currently enrolling dose-escalation study, we are enrolling a control population to compare outcomes.
Blinding	Blinding was not relevant to our study, as there was not a control group. All patients receiving intralesional injections were receiving the study drug (pembrolizumab).

Reporting for specific materials, systems and methods

We require information from authors about some types of materials, experimental systems and methods used in many studies. Here, indicate whether each material, system or method listed is relevant to your study. If you are not sure if a list item applies to your research, read the appropriate section before selecting a response.

Materials & experimental systems

n/a	Involved in the study	
☐	☒	Antibodies
☒	☐	Eukaryotic cell lines
☒	☐	Palaeontology and archaeology
☒	☐	Animals and other organisms
☐	☒	Human research participants
☐	☒	Clinical data
☒	☐	Dual use research of concern

Methods

n/a	Involved in the study	
☒	☐	ChIP-seq
☒	☐	Flow cytometry
☒	☐	MRI-based neuroimaging

Antibodies

Antibodies used	Akoya Opal 7-Color Automation IHC Kit, Lot Number NEL821001KT
Validation	Per Akoya Biosciences website: "The Opal 7-Color Automation Immunohistochemistry Kit is designed for use on the Leica Biosystems BOND RX, and is configured and validated for imaging on the Vectra® and Mantra™ systems."

Human research participants

Policy information about studies involving human research participants

Population characteristics	Patients with pathologically-confirmed breast ductal carcinoma in situ (DCIS) were eligible for enrollment if they possessed at least two of the following high-risk features: young age (< 45 years), large size (> 5cm), high-grade (grade II or III), a palpable mass, hormone receptor (HR) negativity, and/or HER2 positivity.
Recruitment	Participants were recruited from the University of California San Francisco (UCSF) Breast Care Center when evaluated by one of the breast surgical oncologists participating in this study.
Ethics oversight	University of California San Francisco (UCSF) Institutional Review Board

Note that full information on the approval of the study protocol must also be provided in the manuscript.

Clinical data

Policy information about clinical studies

All manuscripts should comply with the ICMJE guidelines for publication of clinical research and a completed CONSORT checklist must be included with all submissions.

Clinical trial registration	NCT 02872025
Study protocol	https://clinicaltrials.gov/ct2/show/NCT02872025?term=02872025&cond=ductal+carcinoma+in+situ&draw=2&rank=1
Data collection	All study participants were recruited at the UCSF Breast Care Center and signed consent between March 2017 and June 2018.
Outcomes	The primary outcome was determination of the maximum tolerated dose of intralesional pembrolizumab while characterizing associated toxicities and assessing feasibility of this novel route of administration. Secondary outcomes included characterization of changes in the tumor immune microenvironment as assessed by multiplex immunofluorescence (mIF), change in tumor cell apoptosis (cleaved-caspase 3 (CC3) staining), change in tumor cell proliferation (Ki67 staining), and change in DCIS volume kinetics by MRI following intralesional pembrolizumab treatment.
